# Is It Possible to Publish a Calibration Function for Radiochromic Film?

**DOI:** 10.4236/ijmpcero.2014.31005

**Published:** 2014-01-29

**Authors:** Maria F. Chan, David Lewis, Xiang Yu

**Affiliations:** 1 Department of Medical Physics, Memorial Sloan-Kettering Cancer Center, Basking Ridge, USA; 2 Advanced Materials Group, Ashland, Inc., Bridgewater, USA

**Keywords:** Dosimetry, Quality Assurance, IMRT, Radiochromic Film, Multichannel

## Abstract

**Purpose:**

To assess the possibility of using a public calibration function for radiochromic film dosimetry in dose QA of highly conformal treatment plans.

**Methods:**

EBT3 film calibration strips (3.5 × 20 cm^2^ from lots A101212 and A011713) were exposed on a Varian Trilogy at a facility to a 10 × 10 cm^2^ open field at doses of 80, 160, 320 cGy using 6MV photons. Together with a strip of unexposed film from the same lot the exposed films were digitized in a single scan using different Epson 10,000 XL scanners at two different facilities. The dose-response data for each color-channel from each facility were generated using the same calibration function X(D) = a + b/(D − c), where X(D) is the response at dose D and a, b and c are the coefficients. Different batches of EBT3 film were exposed to a VMAT beam. These films, plus two reference strips exposed to doses of zero and 160 cGy, were digitized on the scanners at the two facilities. Using the multi-channel dosimetry method and One-scan protocol (*Med Phys*, 39:6339–49, 2012) the recorded doses on the VMAT films were calculated and the results were compared with the VMAT plan using a Gamma index of 3%/3 mm.

**Results:**

The passing rates obtained for dose maps calculated for all combinations of VMAT images and calibration functions were nearly unchanged, using the One-scan protocol. Also, in all cases a passing rate of >99% was obtained for Gamma index of 3%/3 mm. On the other hand, if the One-scan protocol was not employed, the dose maps for VMAT images and calibration functions from different scanners showed poor correlation with the treatment plan. This is probably due to the scan-to-scan variability.

**Conclusions:**

The authors have found that it is feasible to use a public calibration function for a given radiochromic film lot using the same methodology, One-scan protocol, for patient-specific QA.

## 1. Introduction

Radiochromic EBT film has been established as an accurate quantitative 2D dosimeter with fine spatial resolution for applications in external beam and brachytherapy, including small-field dosimetry, IMRT and VMAT quality assurance (QA), commissioning of treatment modalities and verification of treatment planning system (TPS) [1–22]. Since Gafchromic radiochromic films produce colored images when exposed to radiation, it has long been recognized that multichannel flatbed scanners offer better usability than white-light scanners. The red color channel has greater sensitivity at lower doses while the signal from the green or blue channels provides extension of the dynamic range of the film to higher doses [15, 23–25]. Multichannel dosimetry has shown to have significant advantages over single channel dosimetry by its better dosimetric accuracy [26]. A recent publication, Lewis *et al.* [27] raised the possibility of an investigator publishing a dose-response calibration curve for an individual manufacturing lot of EBT2 or EBT3 radiochromic film for use, under specified conditions, by a second user at another location. The requirements for the second user include the use of an Epson flatbed scanner and the adoption of a particular methodology, the “One-scan” protocol [27], involving the scanning of two reference films together with the QA film to be measured. The measured responses of the reference films are used to re-scale the calibration function provided by the first investigator and adapt it to the specific conditions applying to the scan of the second user.

## 2. Materials and Methods

### 2.1. Design of the Study

First, to test the proposal, sets of EBT3 film calibration strips (3.5 × 20 cm^2^ from lots A101212 and A011713) were exposed on a Varian Trilogy at Facility A to a 10 × 10 cm^2^ open field at doses of 80, 160 and 320 cGy using 6MV photons. For exposures the film was placed in a polystyrene phantom with 10 cm of the build-up material above and below the film. The source-to-axis distance (SAD) was 100 cm. Exposure of film for dose calibration was performed with 10 × 10 cm^2^ fields, and the film perpendicular to the axis of the beam. The same polystyrene phantom was used for the exposure of films to VMAT fields. Patient VMAT films were also placed at a depth of 10 cm in the phantom and exposed to the full dose of the treatment plan. Together with a strip of unexposed film from the same production lot the exposed films were digitized in a single scan frame (48-bit *rgb*, 72 dpi) using different Epson 10,000 XL scanners—one at Facility A and one at Facility B. The image processing and film analysis are done using Film QA Pro software (Ashland Inc., Bridgewater, NJ).

The dose-response data for each color channel, from each facility and for each lot number were correlated using the function

**Figure 1:**
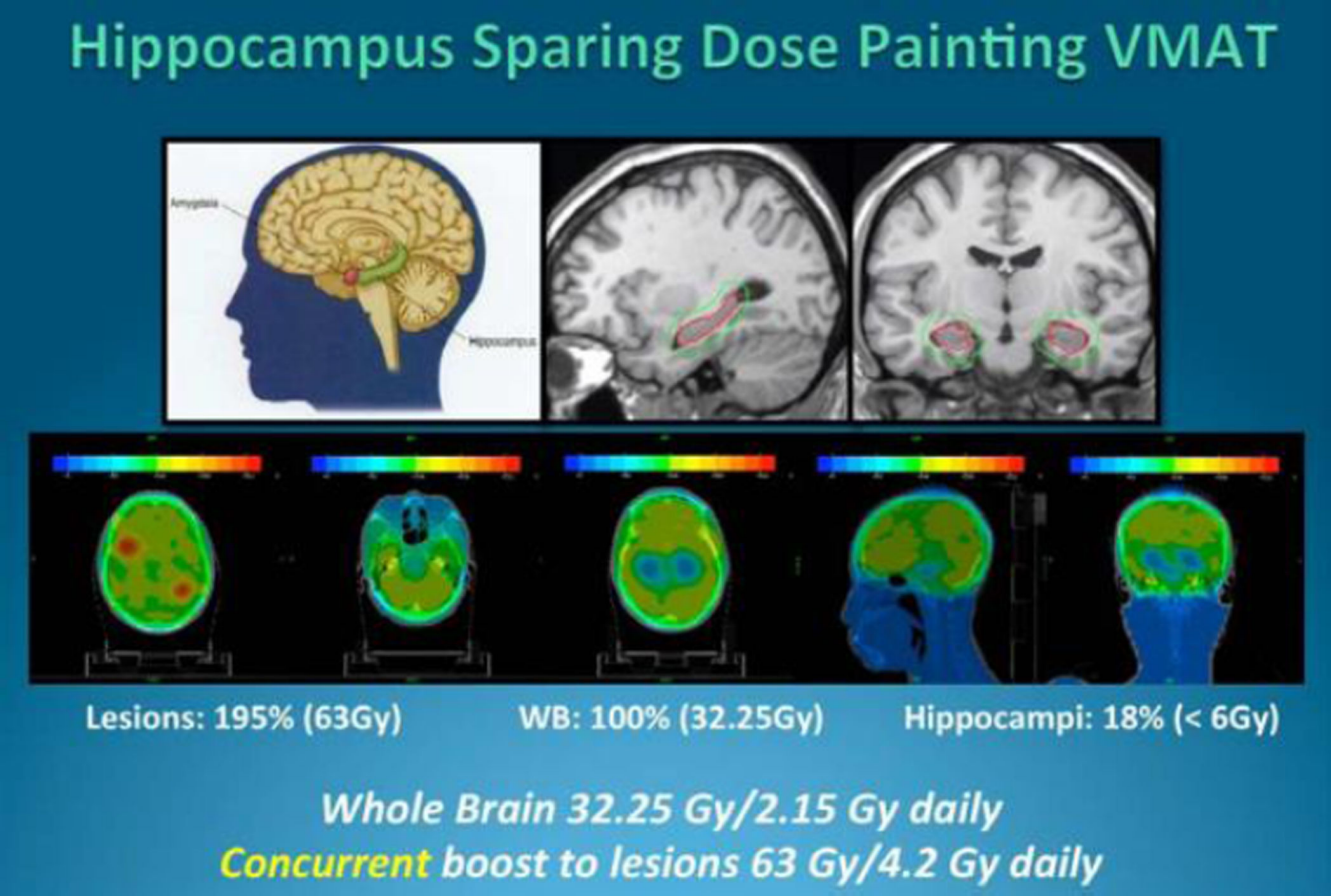
Highly modulated 3 dose levels dose painting VMAT plan was used for the validation study.

**Figure 2:**
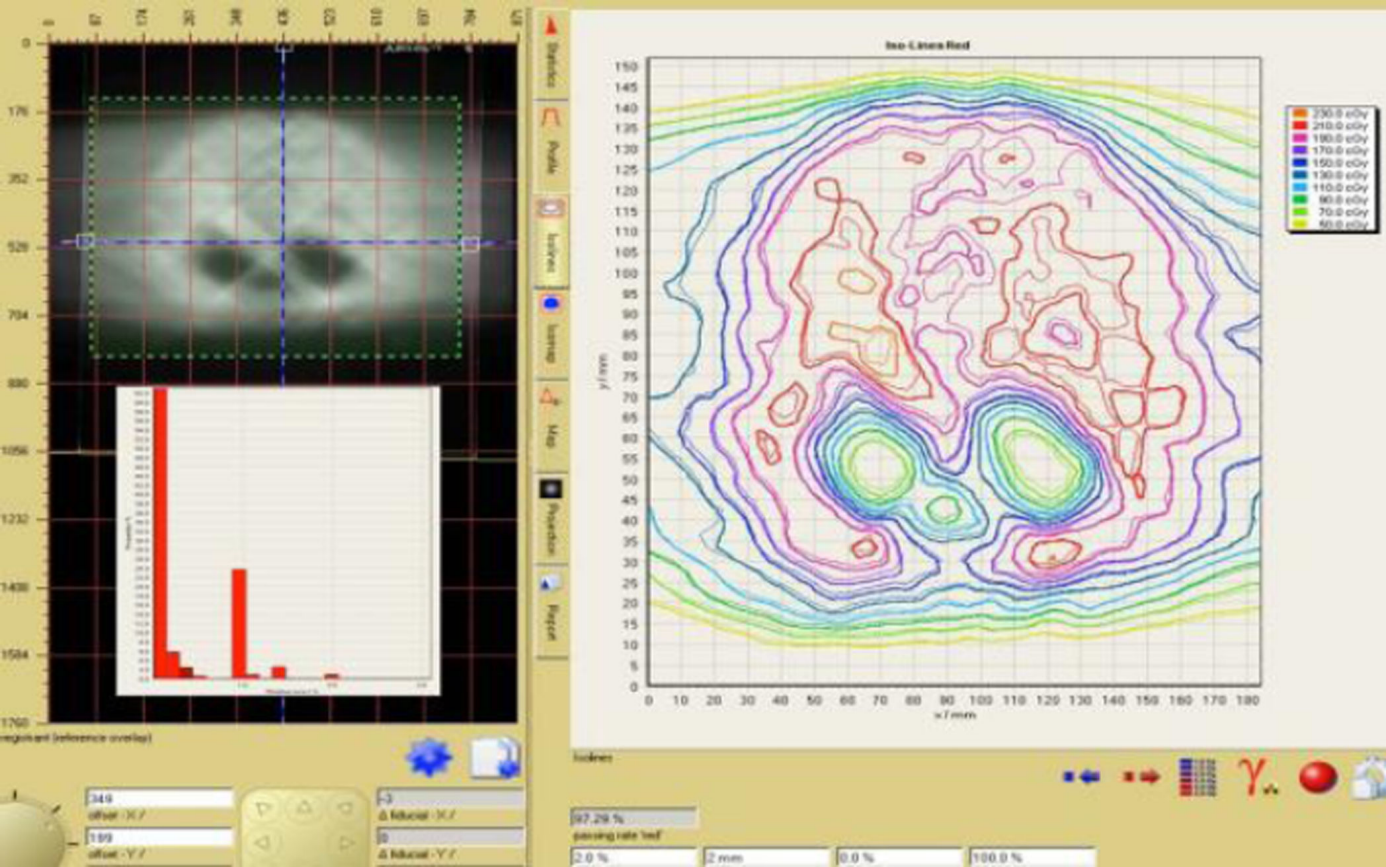
Isodoses overlay between EBT3 film measurement and VMAT plan data in FilmQA Pro.

(1) X(D)=a+b(D−c)

where *X*(*D*) is the response at dose D and a, b and c are the coefficients to be defined. Pieces of EBT3 film from the same production lots were exposed to the single arc of an oligo brain VMAT plan (see Figure 1). These films, plus two reference strips with matching lot numbers exposed to doses of zero and 160 cGy, were digitized on the Epson 10,000 XL scanners at Facilities A and B. Using the multi-channel dosimetry method and “One-scan” protocol the recorded doses on the VMAT films were calculated (see Figure 2) for all combinations of VMAT images and response functions, *i.e.* VMAT image from Facility A with calibration functions from Facilities A and B, etc. The resulting dose maps were projected onto and compared with the VMAT plan using Gamma evaluation and test criteria of 3%/3 mm.

### 2.2. “One-Scan Protocol”- An Original Calibration Curve

**Figure 3:**
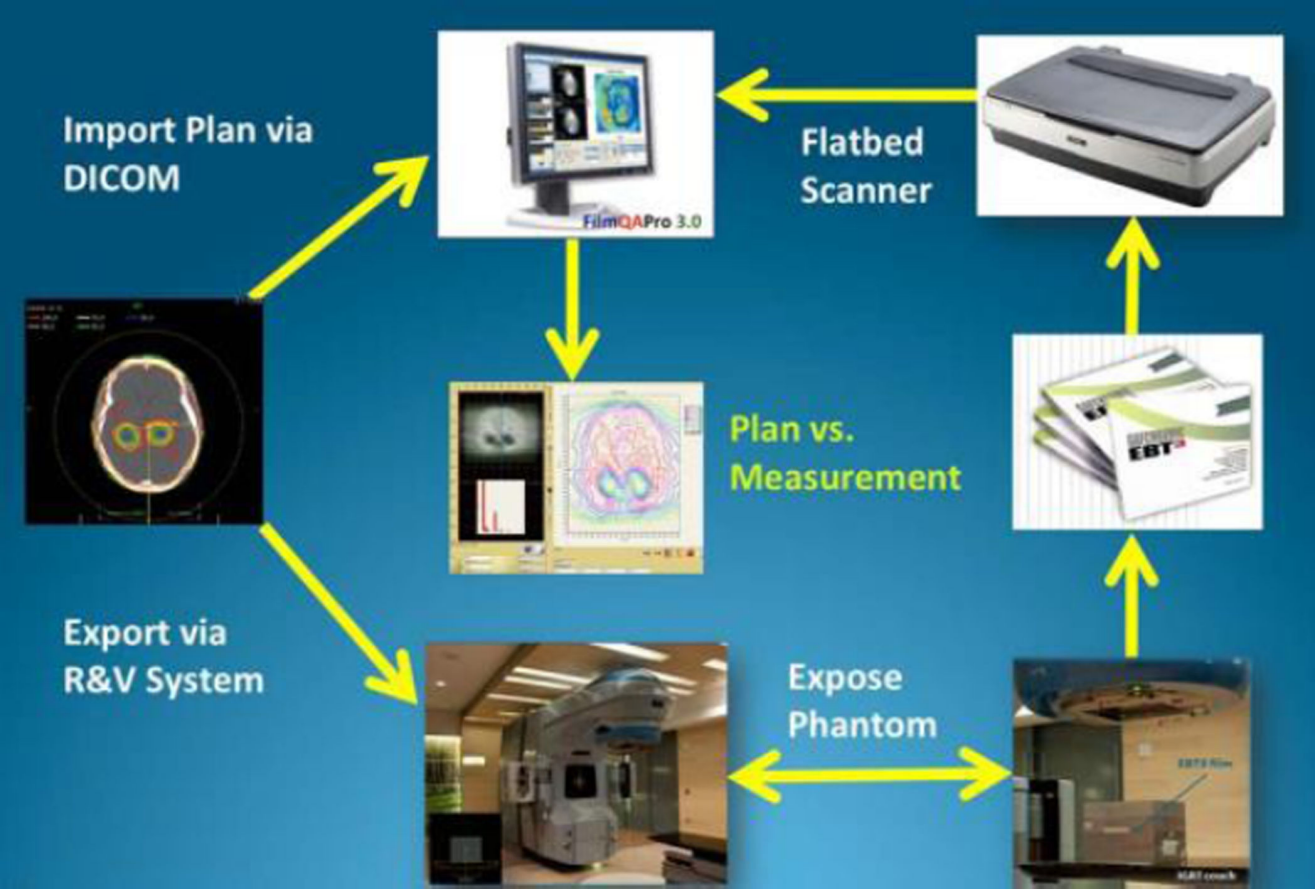
Clinical workflow of the patient-specific VMAT QA.

The dose-response data for a film production lot could be fit to a set of related rational functions leading to the description of a generic calibration curve. A simplified protocol was established where dose-response data for a specific scanner, scanning conditions (time-after exposure, temperature, orientation) and exposure source could be derived from a generic calibration curve using one film exposed to a known dose and an unexposed film to adapt the generic curve to the specific case. The clinical workflow of the film dosimetry is shown in Figure 3.

The normalized response *X* of the system with respect to dose can be correlated using rational functions of the form,

(2) X(D)=A+B(C−D) or X(D)=A+BD(D+C)

where *A, B, C* are parameters that can be fitted to calibration data using least square approach.

For measured data (*n_i_, D_i_*) with = 1(1)I, n normalized system response and D dose, the equation

(3) $$\sum_{i}{\left(N(D_{i})-n_{i}\right)}^{2}\to {\text{min}}_{A,B,C}$$

is minimized to determine the calibration parameters *A, B, C*.

A specific calibration can be derived from the normalized system response N using the rescaling relation

(4) X(D)=α+βN(D)

where *X* is the response in one of the color channels R, G or B. The two parameters α and β can be calculated as

(5) $$\alpha =(N_{1}X_{2}-N_{1}X_{1})(N_{1}-N_{2})$$

(6) $$\text{and }\beta =(X_{1}-X_{2})(N_{1}-N_{2})$$

if two data points (*X_i_, D_i_*), *I* = 1, 2 are available using *N_i_* = *N*(*D_i_*).

**Figure 4:**
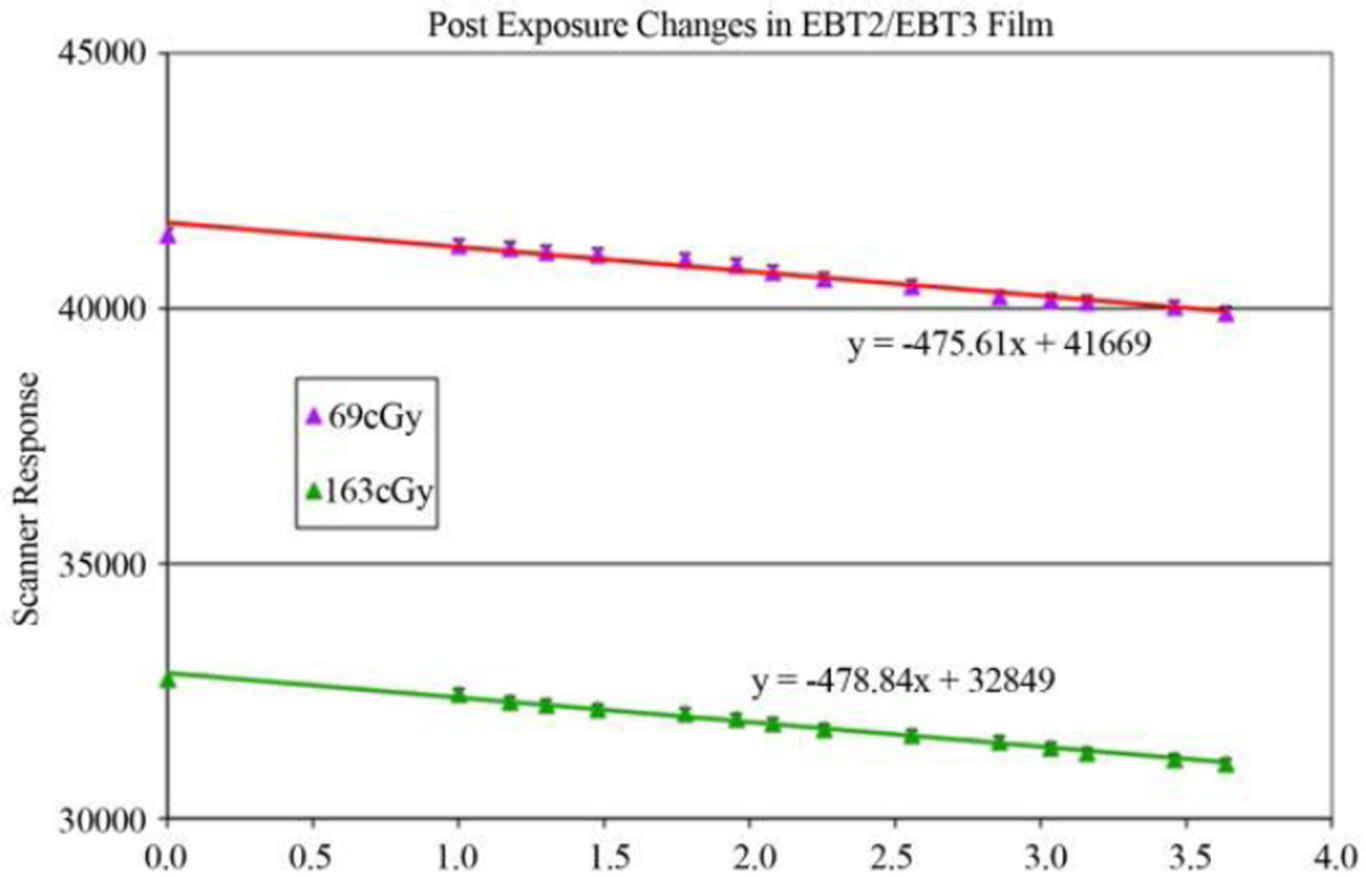
Post exposure changes in EBT2/EBT3 film.

**Figure 5:**
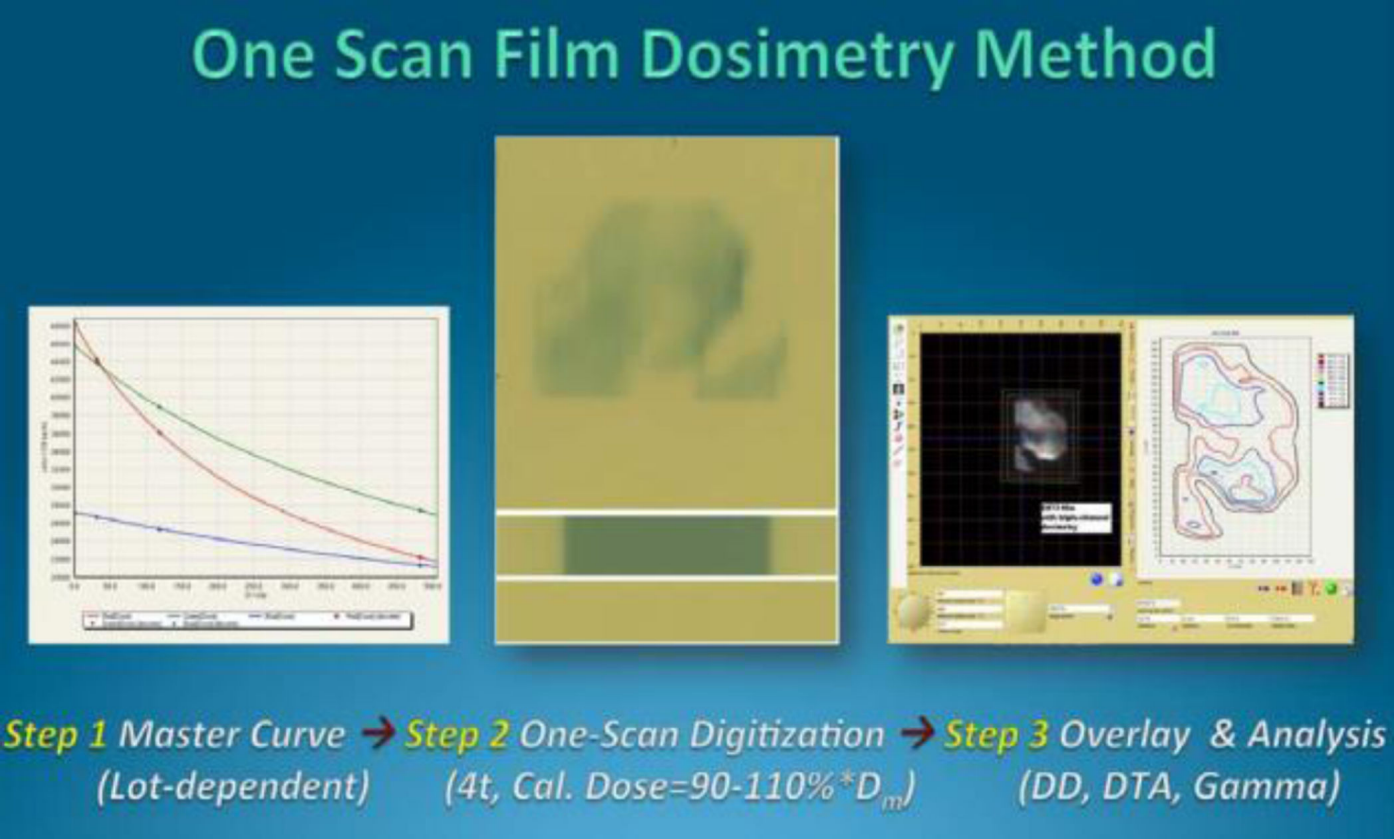
Scheme of “One-scan” protocol using triple-channel film dosimetry.

It is well known that radiochromic film, including the EBT3 film, undergoes post-exposure intensification. In a previous study [27], the authors evaluated the “One-scan” protocol by first collecting dose-response data from six production lots of Gafchromic EBT3 film. The post-exposure changes in Gafchromic EBT3 film response by exposing samples to five doses between 30 and 480 cGy within a 5-minute interval. Together with an unexposed film the samples were digitized in a single scan in 48-bit *RGB* transmission mode on five different Epson 10,000 XL scanners and three different Epson V700 scanners at various elapsed times-after-exposure. The authors measured and report the error due to the timing difference (see Figure 4). The workflow of “One-scan” protocol is shown in Figure 5. This “One-scan” protocol also promises to ease waiting restrictions owing to the well-known post-exposure change in film response to just a few minutes between exposure and scanning. To do this requires the application film to be scanned with two reference films from the same production lot, one reference film unexposed and the other exposed to a dose similar to the highest dose on the application film. To minimize the post-exposure wait before scanning the application and reference films should be exposed within a narrow time window. If the time window is t, the minimum time between exposure and scanning should be 4t to keep dose error <0.5%.

## 3. Results and Discussion

**Figure 6:**
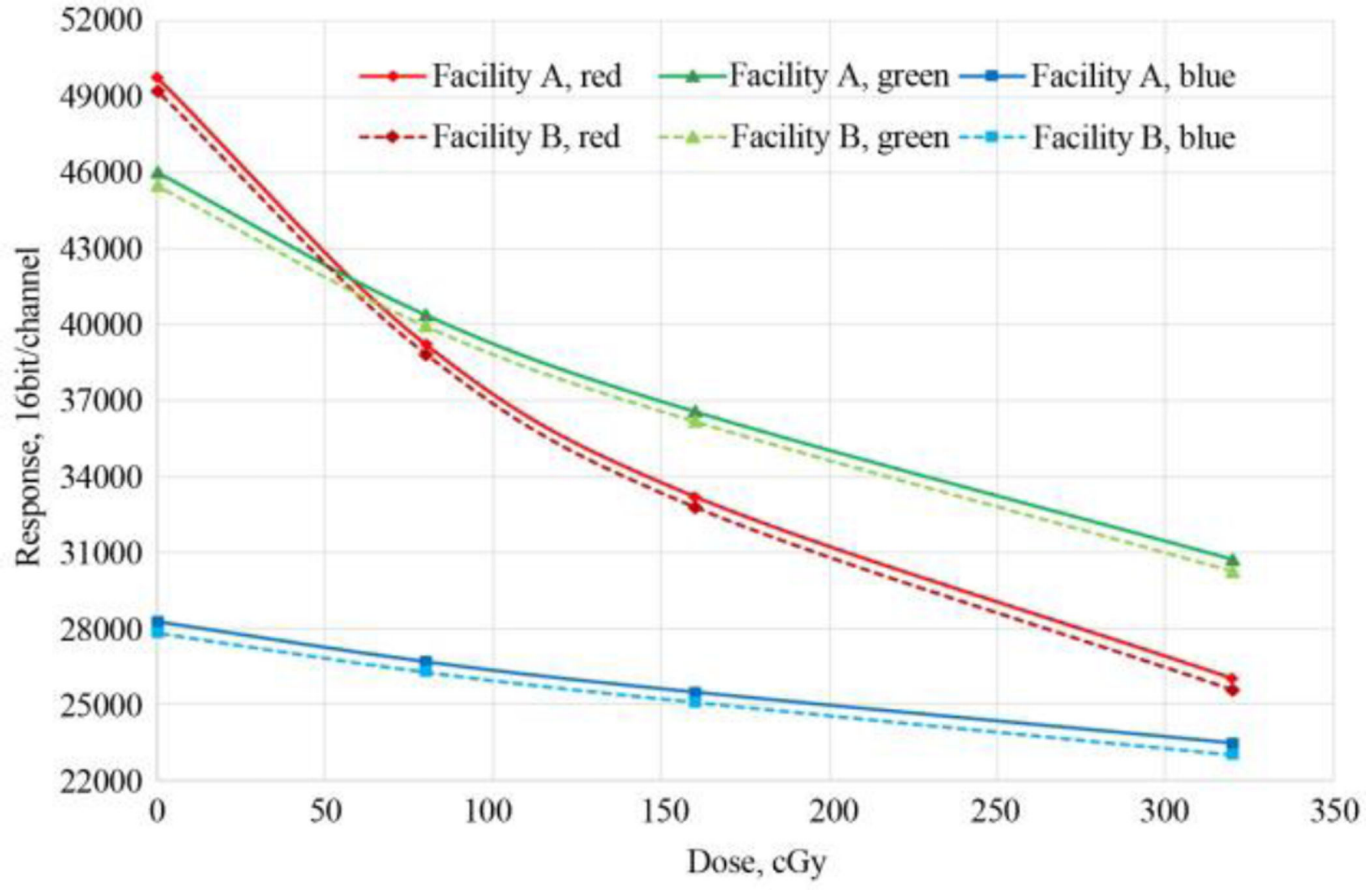
The dose-response data for each color channel from each facility.

A scan image of the calibration film from Facility A was used to calculate a calibration function and applied to calculate dose maps from VMAT film scans in both Facilities. The dose-response data for each color channel, from each facility and for each lot number were correlated using the function (1) and depicted in Figure 6. Using the “One-scan” protocol [27], the passing rates for the dose maps from the two facilities were nearly unchanged with passing rates >99% for a Gamma index of 3%/3mm. Similar results were obtained when the Facility B calibration function was used for calculating the dose maps. In contrast, when a dose map was calculated for the VMAT film scanned at Facility B using the calibration scans at Facility A without the benefit of the “One-scan” protocol and reference films, the calculated map and planned dose distribution were poorly matched with Gamma passing rates <60%. The result reflects the differences in the absolute response values for the same calibration films digitized at the two facilities (see Table 1). However, by using the two reference films and the “One-scan” protocol, the response values from different scanners were rescaled and the resulting dose maps were restored to close agreement with the treatment plan. The authors in a separate study [27] reported that a series of measurements of unexposed EBT3 film taken over 10-days time showed a small response difference correlated with temperature difference, but no pattern of behavior consistent with a permanent change in the film. The differences are most likely due to the inherent stability of the electronic measurement circuits in the scanners as well as small temperature differences from scan-to-scan.

Recently some researchers have demonstrated the use of functional argument to linearize the inherently non-linear response of a radiochromic film based reference dosimetry system [28]. In this way, they showed that relative dosimetry can be conveniently performed using radiochromic film without the need of establishing calibration curve. Then, the authors have subsequently developed a simplified “One-scan” protocol for using radiochromic film that avoids complications encountered in commonly used methods, *i.e.*, multiple-film calibration and multiple-scan image acquisition prior to patient-specific QA in order to obtain absolute dose values. Together with the triple-channel radiochromic media dosimetry method [26], response curve linearization of the radiochromic film dosimetry system [28], the “One-scan” protocol [27], and now the public calibration function indicates another significant advancement in radiochromic technology, design, and function for streamlining of patient-specific IMRT QA.

The energy dependence of the EBT/EBT2/EBT3 film response induced by different radiation beam qualities has been investigated by various research groups [29–35]. The EBT2 film response to nine energy X-ray beams between 50 kV and 10 MV has been investigated and an energy dependence of about 6.5% in the optical density per unit dose measured in the entire energy range by analyzing the red component was reported [31]. Such a result was supported by independent research study [32] comprising kilovoltage X-rays (75, 125, and 250 kV), ^137^Cs and ^60^Co Gamma, megavoltage X-rays (6 and 18 MV), electron beams (6 and 20 MeV) and proton beams (100 and 250 MeV), where the energy dependence of EBT2 was found to be relatively small within measurement uncertainties (4.5%) for all energies and modalities [32]. In contrast, other study reported variation up to 20% on the energy dependence of EBT2 film for photon energy between 105 kV and 6 MV, depending on the batch number, which was interpreted as a consequence of variation in the concentrations of bromine, chlorine, and potassium among batches [33]. This result is in agreement with Monte Carlo simulation where the EBT2 film’s response to energy photon below 100 keV was found to be energy dependent of about 10% and 50%, depending on the manufacturing lot, due to changes in the ratio of mass energy absorption coefficients of the active emulsion layers to water [34]. Thus, given the non-universality on the energy dependence of the EBT2 film response to energy photons. More recently the new EBT3 film has emerged to the market, researchers [35] have evaluated the energy dependence of the new EBT3 film with 50 kV, 6 and 15 MV X-ray beams. It was found that the film’s response is weakly dependent on the energy of high-energy photon beams generally used in radiotherapy; however, for very low-energy photon (e.g. 50 kV), variation of more than 11% due to the energy-dependence is observed. Thus, for brachytherapy seeds like ^125^I and ^103^Pd, special attention is required in calibrating the film response to low energy photons.

A public calibration function was demonstrated and validated through VMAT dose QA scanned at two different facilities wherein a film to be measured and check films were exposed within a narrow time window and then scanned together at the same time. This procedure simplifies radiochromic film dosimetry and speeds its application for patient-specific IMRT and MVAT plan verification. Since IMRT and check films are scanned together, interscan variability is eliminated as a source of error. As good results were obtained from calibration data acquired under different conditions the protocol accepts the use of a generic calibration function. The uncertainties of the measured doses were estimated following the method described in the EBT3 film studies [6,11]. Combining the Type A (statistical) and Type B (non-statistical) uncertainties, the uncertainties of the measured doses at individual pixels were estimated to be 2% as compared to ~4% for the traditional single-channel film dosimetry.

## 4. Conclusion

The authors have found that it is feasible to use a public calibration function for patient-specific IMRT/VMAT QA for a given radiochromic film lot using the reference film methodology and the “One-scan” QA protocol, in conjunction with the “One-scan” film dosimetry protocol. This further simplifies the QA process and provides a practical solution for using radiochromic film for routine patient-specific dose verification without sacrificing spatial resolution.

## Figures and Tables

**Table 1:** Gamma evaluation of VMAT and calibration films scanned at two different facilities.

One-Scan Protocol with Reference Films	Scanner Location		Gamma Evaluation—3%/3 mm % Pixels Passing	

	Calibration Scan	VMAT Scan	Film Lot A101212	Film Lot A011713
Yes	Facility A	Facility A	99.5	99.5
Yes	Facility A	Facility B	99.2	99.6
No	Facility A	Facility B	59.2	55.1
Yes	Facility B	Facility A	99.3	99.4
Yes	Facility B	Facility B	99.7	99.7
